# Hepatitis C virus modelled as an indirectly transmitted infection highlights the centrality of injection drug equipment in disease dynamics

**DOI:** 10.1098/rsif.2019.0334

**Published:** 2019-09-04

**Authors:** Miles D. Miller-Dickson, Victor A. Meszaros, Salvador Almagro-Moreno, C. Brandon Ogbunugafor

**Affiliations:** 1 Department of Ecology and Evolutionary Biology, Brown University, Providence, RI 02906, USA; 2 Burnett School of Biomedical Sciences, University of Central Florida, Orlando, FL 32827, USA; 3 National Center for Integrated Coastal Research, University of Central Florida, Orlando, FL 32816, USA

**Keywords:** mathematical modelling, epidemiology, ecology of infectious disease

## Abstract

The hepatitis C virus (HCV) epidemic often occurs through the persistence of injection drug use. Mathematical models have been useful in understanding various aspects of the HCV epidemic, and especially, the importance of new treatment measures. Until now, however, few models have attempted to understand HCV in terms of an interaction between the various actors in an HCV outbreak—hosts, viruses and the needle injection equipment. In this study, we apply perspectives from the ecology of infectious diseases to model the transmission of HCV among a population of injection drug users. The products of our model suggest that modelling HCV as an indirectly transmitted infection—where the injection equipment serves as an environmental reservoir for infection—facilitates a more nuanced understanding of disease dynamics, by animating the underappreciated actors and interactions that frame disease. This lens may allow us to understand how certain public health interventions (e.g. needle exchange programmes) influence HCV epidemics. Lastly, we argue that this model is of particular importance in the light of the modern opioid epidemic, which has already been associated with outbreaks of viral diseases.

## 1. Introduction

While the ecology of infectious disease is a rich field with decades worth of empirical evidence and theory, there are aspects that remain relatively under-explored. One example is the importance of the free-living survival stage of certain pathogens, where diseases are transmitted indirectly between hosts through an environmental reservoir intermediate. These include infections transmitted indirectly between hosts via a surface or reservoir intermediate—often abiotic—where the pathogen lives freely and independently of a host [1–18], sometimes described as ‘sit and wait’ infections [19]. Other studies have focused on systems where pathogens are growing in the environment [9], or have explored indirectly transmitted infections in theoretical terms [12,15]. While frameworks already exist for studying indirect environmental transmission, most are engineered with constraints that render their application necessarily narrow [6], limiting their relevance for a wider number of indirectly transmitted infections.

One class of diseases where the indirect transmission paradigm has been scarcely applied are those spread through injection drug use in urban settings, such as the human immunodeficiency virus (HIV) and hepatitis C virus (HCV). HIV has been the object of many important mathematical models [20,21], some of which have implemented injection drug use effectively, even focusing on the specific dynamics of injection equipment [22–25]. HCV has also been studied using modelling methods, many focusing on treatment [26–28] and others on the particulars of transmission in injection drug-user communities [29–35]. Importantly, none of these existing dynamical models consider the peculiar ecology of HCV transmission, where transmission events occur through an environmental reservoir (injection equipment) that resembles a disease vector [36,37]. Unlike an insect vector, however, injection equipment is not an organism and is more realistically considered an abiotic reservoir for infection, similar to the role that the water supply serves in an outbreak of cholera or other waterborne diseases [38]. As HCV continues to pose a serious public health challenge in many communities, there is a need to understand how the dynamics of injection equipment influence HCV transmission. This is especially important for informing the utility of harm reduction programmes, such as needle exchange, which have been effective in decreasing transmission of HIV and HCV [39,40]. Lastly, but perhaps most importantly, the urgency for understanding these dynamics has increased dramatically in recent years with the growth of the modern opioid epidemic, much of it involving injection drug use [41,42]. The lack of models of HCV that specifically consider injection equipment, and increased social urgency related to the modern opioid epidemic implore more adaptable mathematical models of injection-drug use that could facilitate a better understanding of and predictions for the trajectory of modern HCV infections.

In this study, we model HCV as an indirectly (or environmentally) transmitted infection, where the drug paraphernalia serves as the environmental reservoir. As HCV epidemics are partly defined by injection drug users and injection drug equipment, we argue that this indirectly transmitted lens captures aspects that prior models have not. In §2, we introduce a theoretical iteration of an indirectly transmitted infection using a standard epidemiological model imbued with an environmental reservoir compartment. We describe analytic equations of such a system, and derive the reproductive number (*R*_0_) using analytic methods. Then in §3, we introduce the full HCV mathematical model, demonstrating how it allows one to examine several otherwise-overlooked features of disease dynamics. We pontificate on these results in light of the ecology of infectious diseases, and in terms of public health policies, especially as they relate to the modern opioid epidemic.

## 2. An elementary adapted SIR indirectly transmitted iteration

### 2.1. Description

While the emphasis of our examination will reside in how we analyse a HCV epidemic, for explanatory purposes we will begin by describing how an environmental reservoir modifies very basic concepts in a classic, purposefully prosaic susceptible–infected–recovered (SIR) mathematical model. We will explain the basic structure of a model of indirect transmission, after which the HCV-specific iteration will be discussed.

While there are several existing frameworks that can be used to describe infections spreading through an environmental reservoir, we have conveniently labelled ours the waterborne, abiotic and indirectly transmitted (W.A.I.T.) infection model. Many diseases can be modelled using this kind of approach, but this study applies it to HCV in a community of injection drug users, which has not been previously modelled in this manner.

**Figure 1.**
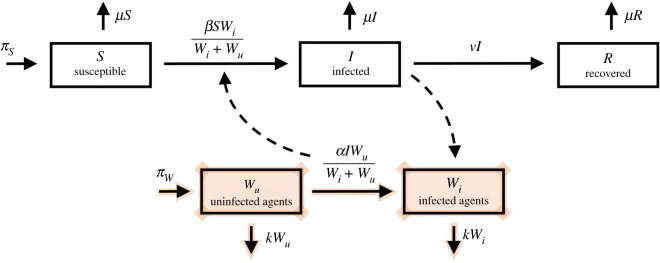
Adapted SIR compartmental diagram. This depicts a standard SIR style compartmental model with the added compartments (shaded) corresponding to the W.A.I.T. environment. Note the dynamical properties of the *W*_*i*_ and *W*_*u*_ compartments. (Online version in colour.)

We use a standard SIR framework, where dynamics are defined by changes in a population of susceptible (S), infected (I) and recovered (R) hosts. Classically, flow of infection through the system is defined by contact between susceptible and infected individuals, often driven by a *β* factor, or transmission coefficient. Figure 1 is a compartmental model that depicts this interaction, and adds two additional compartments, labelled with a *W* (for W.A.I.T.), which influence the flow of hosts from the susceptible to infected compartments—indicated by the dashed lines in the figure.

### 2.2. The adapted SIR compartmental diagram

The *S*, *I* and *R* compartments represent the usual *susceptible*, *infected* and *recovered* populations of hosts, respectively. *W*_*u*_ and *W*_*i*_ represent uninfected and infected populations of *environmental* agents, respectively.

In traditional SIR models, the rate of new infection (arrow from the *S* compartment to the *I*) is generally proportional to the product of the susceptible and the infected populations, i.e. proportional to *SI*. In the W.A.I.T. framework, the environmental compartment plays a role analogous to the infected *host* compartment in driving the rate of infection. In this specific example, the *W*_*i*_ compartment contributes to the rate of infection as a fraction, *W*_*i*_/(*W*_*i*_ + *W*_*u*_), which appears as a factor in the rate terms.

The epidemic is then driven by a series of interactions: between uninfected (susceptible) hosts *S* and the infected (transmitting) environmental compartment *W*_*i*_, and interactions between infected individuals *I* and the uninfected environmental compartment *W*_*u*_. The epidemic is sustained through infected hosts *I* depositing pathogen into the environmental reservoir, creating new infections, which can then infect more susceptible hosts *S* (in a process resembling a feedback loop). These dynamics can be captured by the set of dynamical equations and visualized with the diagram in figure 1:

2.1 $$\frac{dS}{dt}=\pi_{S}-\beta S\frac{W_{i}}{W_{u}+W_{i}}-\mu S,$$

2.2 $$\frac{dI}{dt}=\beta S\frac{W_{i}}{W_{u}+W_{i}}-\nu I-\mu I,$$

2.3 $$\frac{dR}{dt}=\nu I-\mu R,$$

2.4 $$\frac{dW_{u}}{dt}=\pi_{W}-\alpha I\frac{W_{u}}{W_{u}+W_{i}}-kW_{u}$$

2.5 $$\mathrm{and}\;\frac{dW_{i}}{dt}=\alpha I\frac{W_{u}}{W_{u}+W_{i}}-kW_{i}.\;$$

Equations (2.1)–(2.5) define an extension of the prosaic SIR model. *π*_*S*_ is the birthrate of new susceptible hosts and *μ* is the fractional death rate of hosts. In this context, *β* represents the *strength* of the interaction between the susceptible hosts *S* and the environmental reservoir. This will generally be proportional to the rate of contact between the two. Similarly, *α* characterizes the strength of interaction between infected hosts *I* and the environmental reservoir, and is also generally proportional to the contact rate between the two. *α* and *β*, while both generally proportional to the contact rate between environmental agents and living hosts, are distinguished by factors that indicate the probabilities of spreading the infection either from host to environment, as in the case of *α*, or from the environment to host, as in the case of *β*. Note that *α* and *β* could be replaced with the same parameter in settings where the infection is guaranteed to spread at any encounter with an infected host or environmental agent. *ν* represents the fractional recovery rate, *π*_*W*_ is the birthrate of new uninfected environmental agents and *k* is the fractional death or discard rate of environmental agents. Note that the discard rate *k* (which includes any force that removes environmental agents from the system) can be split into two discard rates, one for the infected compartment, and one for the uninfected compartment (we do, in fact make this distinction in the full HCV model). For simplicity, we will tend to set the discard rates of these two compartments equal, where there are two parameters, as we have no current mathematical grounds to distinguish them.

Also note that this model resembles vector-borne transmission models such as those used to study malaria [37]. In fact, prior studies have explored the utility of applying vector-borne transmission models to the spread of infection with needles as a proxy for vectors—although, only in the context of HIV [36]. In this paper, however, we wish to emphasize peculiarities of the spread of HCV and, further, to elaborate some features of the *R*_0_ expression in the context of these *shared* dynamical settings—between hosts and agents—which we believe have not been rigorously addressed in the existing literature.

### 2.3. W.A.I.T. framework influences the basic reproductive number in a standard SIR model

Next, we briefly consider how the value of the basic reproductive ratio *R*_0_ in this model compares to its SIR counterpart. While *R*_0_ can have different theoretical formulations, we rely on definitions as provided by Jones [43] and Diekmann *et al*. [44]. In a density-dependent SIR model with constant birth of susceptible hosts *π*_*S*_ and death rate proportional to the host population −*μS*, the *R*_0_ value is given by

2.6 $$R_{0}^{SIR}=\frac{\beta \pi_{S}}{\nu \mu },$$

or sometimes, more simply, $R_{0}^{SIR}=\beta /\nu$, depending on the form of the *SIR* equations used, e.g. frequency-dependent, constant population, etc. In this equation, *β* is the traditional transmission coefficient. It represents the coupling strength between infected and uninfected hosts, two non-environmental agents. Whereas, in the W.A.I.T. model, what is analogous to *β* is a pair of parameters *α* and *β*, which govern the interaction strengths between hosts and the environment. *π*_*S*_, *μ* and *ν* have the same interpretation as in the W.A.I.T. model.

In the case of the W.A.I.T. iteration, the value of *R*_0_ takes the form

2.7 $$R_{0}^{\mathrm{WAIT}}=\sqrt{\frac{\alpha \beta \pi_{S}}{\mu (\mu +\nu )\pi_{W}}}.$$

There are some notable differences in the *R*_0_ formulae of the SIR and W.A.I.T. models: the square root in the W.A.I.T. version arises as a consequence of implementing two infected agents (*I* and *W*_*i*_) into the model, as opposed to just one in the SIR case. Next, one notices that the *β* factor in the SIR formula is augmented by the additional factor *α* in the W.A.I.T. formula, representing a kind of shared dependence between the couplings controlling the *I*-interaction (*α*) and the *S*-interaction (*β*) with the environment. Analogously, what was the responsibility of *π*_*S*_ in the SIR formula now presents itself as a shared dependence, *π*_*S*_/*π*_*W*_, the ratio of the birthrate of susceptible hosts to that of uninfected environmental agents. In this case, the two appear as a ratio under the square root, as opposed to a product in the *αβ* case, indicating that whereas *α* and *β* contribute to *R*_0_ in the same way, *π*_*S*_ and *π*_*W*_ contribute in opposite ways: when *π*_*S*_ is increased, *R*_0_ increases, but when *π*_*W*_ is increased, *R*_0_ decreases.

It is possible to view $R_{0}^{\mathrm{WAIT}}$ as a geometric mean of two *R*_0_ values. Namely, there is the reproductive ratio associated with the number of secondary host infections caused by a single infected environmental agent, and there is the reproductive ratio associated with the number of secondary environmental agent infections caused by a single infected host. We denote the former by $R_{0}^{H}$ and the latter by $R_{0}^{W}$ (*H* for hosts and *W* for the W.A.I.T. compartment). From equations (2.1)–(2.5), one can see that the rate of new host infection due to infected environmental agents *W*_*i*_ is given by *βSW*_*i*_/(*W*_*i*_ + *W*_*u*_). Near the disease-free equilibrium (DFE), *S* ≈ *π*_*S*_/*μ* and *W*_*i*_/(*W*_*i*_ + *W*_*u*_) ≈ *kW*_*i*_/*π*_*W*_ (near the DFE, *W*_*i*_ ≪ *W*_*u*_), which implies that near the DFE, the rate of new host infection per *infected* environmental agent is ≈*βπ*_*S*_*k*/(*μπ*_*W*_). The average amount of time an infected environmental agent remains infected is 1/*k*, i.e. the reciprocal of the exit rate of the infected state. Thus, the number of new host infections caused by an infected environmental agent in the time that the agent is infected, and while the system is near the DFE, is given by *βπ*_*S*_*k*/(*μπ*_*W*_) × 1/*k* = *βπ*_*S*_/(*μπ*_*W*_). That is

2.8 $$R_{0}^{H}=\frac{\beta \pi_{S}}{\mu \pi_{W}}.$$

Similarly, the rate of new infection of environmental agents, caused by infected hosts, is given by *αIW*_*u*_/(*W*_*i*_ + *W*_*u*_). Near the DFE, this rate, per infected host, is ≈*α* (since *W*_*u*_/(*W*_*i*_ + *W*_*u*_) ≈ 1), and the average time that an infected host remains infected is given by 1/(*μ* + *ν*), the reciprocal of the exit rate of the infected state. Thus, the number of new environmental agent infections caused by an infected host in the time that the host is infected (near the DFE) is given by

2.9 $$R_{0}^{W}=\frac{\alpha }{\mu +\nu }.$$

One can see that the value of *R*_0_ given in equation (2.7) is the geometric mean of the two *R*_0_ values calculated above:

2.10 $$R_{0}^{\mathrm{WAIT}}=\sqrt{\frac{\beta \pi_{S}}{\mu \pi_{W}}}\times \sqrt{\frac{\alpha }{\mu +\nu }}=\sqrt{R_{0}^{H}R_{0}^{W}}.$$

From this perspective, one can observe how a characteristic feature of the epidemic is modified by *indirect* transmission.

## 3. The hepatitis C virus model

### 3.1. Description

Our HCV model represents an adaptation of the SIR W.A.I.T. model outlined in §2, but engineered around the particulars of HCV. Our model simulates a population of approximately 170 000 individuals—based on estimates of the size of the people who inject drugs (PWID) community in New York City [45]—where infected injection drug users may migrate into the population. In this model, injection paraphernalia serve as the environmental reservoir for HCV and the sharing of this equipment will constitute the means of transmitting new infections. While the entirety of injection paraphernalia might contain other components, many parameters in this model are based on the use of needle and syringe as the instrument of injection and sharing. Consequently, we use the term ‘needle’ in this paper as a synecdoche for the entire injection apparatus. It is also important to note that HCV can be transmitted sexually [46], but in this study we restrict our attention to transmission through infected needles. This main text focuses on the main structure and dynamical properties of the model. Further model details and discussion can be found in the electronic supplementary material, appendix.

### 3.2. HCV W.A.I.T. model: compartmental diagram

**Figure 2.**
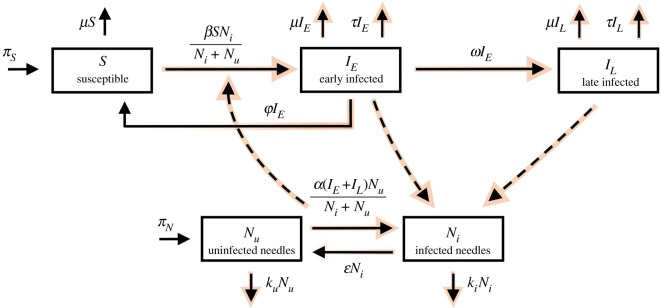
HCV compartmental diagram. Red arrows highlight flow of disease through the system, and where there is a colour/transparency gradient there is a flow of infection away from an infected compartment towards an uninfected one. (Online version in colour.)

We model the dynamics of needle populations and injection drug users through a series of five ordinary differential equations. The compartments labelled *S*, *I*_*E*_, *I*_*L*_, *N*_*u*_ and *N*_*i*_ represent the populations of susceptible individuals, early-stage infected individuals (acute HCV infection), late-stage-infected individuals (chronic HCV infection), uninfected needles and infected needles, respectively (figure 2). Here, we refer to all needles in circulation within the entire PWID community. This model is defined by several features:

— The susceptible compartment refers to individuals who are injecting drugs and who are sharing needles with other members in the PWID community.
— The needle population is divided into two compartments: infected and uninfected, and we model the dynamics of each compartment separately. This is analogous to the *W*_*i*_ and *W*_*u*_ terms discussed in the preliminary model.
— New infections (of both hosts and needles) will depend on the *fraction* of infected or uninfected needles in circulation.
— Newly infected individuals enter the early stage compartment *I*_*E*_ first before either spontaneously clearing the infection or moving into the late-stage compartment *I*_*L*_, from which we assume no spontaneous clearance occurs—individuals may leave *I*_*L*_ either by treatment or death only, since cases of spontaneously clearing *chronic* HCV are rare.
— There are various estimates for the ability of HCV to survive in needles [47,48]. We incorporate HCV free-living survival via the parameter *ε*, which quantifies the rate at which the virus decays on infected needles.

### 3.3. HCV W.A.I.T. model: analytic equations and parameters

The dynamics of the HCV transmission process are governed by equations (3.1)–(3.5). The population of individuals that are being treated and those who have recovered are not explicitly modelled in this iteration, as the dynamics of treatment and recovery are not central to the questions explored in this study. There are, however, several modelling studies of HCV that focus on treatment [26–28,49], and their effects are not ignored in the HCV W.A.I.T. model.

Entering treatment (and re-entering the susceptible population, as in case of drug relapse in the PWID population) are incorporated via removal terms −*τI*_*L*_ and −*τI*_*E*_ and the susceptible ‘birth’ term *π*_*S*_.

3.1 $$\frac{dS}{dt}=\pi_{S}+\phi I_{E}-\beta S\frac{N_{i}}{N_{i}+N_{u}}-\mu S,$$

3.2 $$\frac{dI_{E}}{dt}=\beta S\frac{N_{i}}{N_{i}+N_{u}}-(\omega +\tau +\mu +\phi )I_{E},$$

3.3 $$\frac{dI_{L}}{dt}=\omega I_{E}-(\mu +\tau )I_{L},$$

3.4 $$\frac{dN_{u}}{dt}=\pi_{N}-\alpha (I_{E}+I_{L})\frac{N_{u}}{N_{i}+N_{u}}-k_{u}N_{u}+\epsilon N_{i}$$

3.5 $$\mathrm{and}\;\frac{dN_{i}}{dt}=\alpha (I_{E}+I_{L})\frac{N_{u}}{N_{i}+N_{u}}-k_{i}N_{i}-\epsilon N_{i},\;\;$$

where *π*_*S*_ is the birthrate of new members into the PWID community either via migration, first-time use or recovery *from* treatment—not from spontaneous *self-clearance*. *ϕ* represents the daily fractional rate that acutely infected individuals (or *early infected* *I*_*E*_) spontaneously clear the infection—i.e. without treatment. *α* represents the *per capita* injection rate, scaled by the fraction of injection events by infected users that render a needle infectious. *β* represents the *per capita* injection rate, scaled by the fraction of injection events with an infected needle that leave a susceptible host infectious. *μ* is the combined fractional death and PWID cessation rate (individuals who leave the PWID community). *ω* is the daily fractional rate that early stage infected individuals progress to the late stage of infection. *τ* is the daily fractional rate that infected individuals go into treatment. *π*_*N*_ is the rate of introduction of uninfected needles into the PWID population. *k*_*u*_ is the daily fractional discard rate of uninfected needles. *k*_*i*_ is the daily fractional discard rate of infected needles. Lastly, ϵ is the daily fractional rate that infected needles clear the infection due to de-activation (or ‘death’) of virus populations on the needle. Parameter values and sources can be seen in table 1.

**Table 1.** HCV model parameters.

label	value	units	description	sources
*π*_*S*_	47 ± 10	person/day	birthrate of susceptibles (chosen to keep *π*_*N*_/*μ* ≈ 170 000)	estimate
*ϕ*	(4.7 ± 0.5) × 10^−3^	%/day	daily fractional self-clearance rate	[26,50]
*α*	4 ± 3	$\frac{\mathrm{injections}}{\mathrm{person}\cdot \mathrm{day}}$	injection rate times infection of needle probability	[51]
*β*	0.072 ± 0.05	$\frac{\mathrm{injections}}{\mathrm{person}\cdot \mathrm{day}}$	injection rate times infection of host rate	[52]
*μ*	(2.7 ± 0.5) × 10^−4^	%/day	fractional rate of removal from PWID community due to cessation and death	[53]
*ω*	0.006 ± 0.005	%/day	fractional transfer rate into late-stage infection	[54,55]
*τ*	0.011 ± 0.005	%/day	fractional rate of entering treatment	[56,57]
*π*_*N*_	(3.14 ± 0.01) × 10^4^	needles/day	birthrate of uninfected needles (chosen to keep *π*_*N*_/*k*_*u*_ ≈ 220 000)	[58,59]
*k*_*u*_	0.143 ± 0.005	%/day	fractional discard rate of uninfected needles	estimate
*k*_*i*_	0.143 ± 0.005	%/day	fractional discard rate of infected needles	estimate
*ɛ*	1.17 ± 0.05	%/day	fractional decay rate of HCV infection in needles	[48]

### 3.4. HCV W.A.I.T. model parameters influence *R*_0_

Having constructed and elaborated on the details of the HCV W.A.I.T. model, we now explore how parameters related to the environmental reservoir (in this case, those framing the population of infected needles) influence *R*_0_. We directly measured the influence of parameters on *R*_0_ by considering the *partial rank correlation coefficient* (PRCC), discussed below. The value of *R*_0_ was calculated using established methods [43,44] and is outlined in the electronic supplementary material, appendix:

3.6 $$R_{0}=\sqrt{\frac{\alpha \beta k_{u}\pi_{S}(\mu +\tau +\omega )}{\pi_{N}\mu (\epsilon +k_{i})(\mu +\tau )(\mu +\tau +\phi +\omega )}}.$$

We emphasize that in a manner analogous to our example discussed in §2, we can regard our *R*_0_ value as a geometric mean of two other *R*_0_ values:

3.7 $$R_{0}=\sqrt{\frac{\alpha (\mu +\tau +\omega )}{\left(\mu +\tau )(\mu +\tau +\phi +\omega \right)}}\times \sqrt{\frac{\beta k_{u}\pi_{S}}{\mu (\epsilon +k_{i})\pi_{N}}}.$$

The left-most factor (under the square root) can be interpreted as the number of secondary infections of *needles* in the average time that a *host* is infected (near the DFE), and the right-most factor can be regarded as the number of secondary infections of *hosts* in the average time that a *needle* remains infected. Further discussion of this result can be found in the electronic supplementary material, appendix. As with traditional values of *R*_0_, we find that our value is consistent with the statement that sign(*R*_0_ − 1) = sign(*λ*), where *λ* is the maximal eigenvalue of the Jacobian of the infected subsystem—composed of the infected compartments of the ODE system: *I*_*E*_, *I*_*L*_ and *N*_*i*_—calculated at the DFE (all eigenvalues of the Jacobian were real-valued). This shows that the DFE is unstable when *R*_0_ > 1.

**Figure 3.**
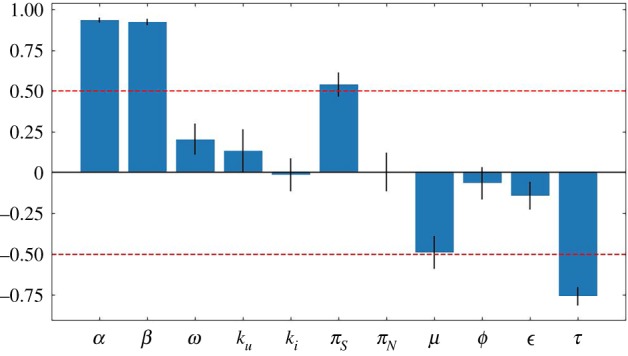
*R*_0_ sensitivity in HCV: the partial rank correlation coefficient (PRCC). A PRCC calculation was performed for *R*_0_ using Latin hypercube sampling. Parameters were sampled from uniform distributions with widths specified by the ranges given in table 1. The PRCC calculation was repeated for 50 independent iterations. The averages of these iterations are shown here, with the standard deviations for each parameter shown as the error bars. (Online version in colour.)

We determine the sensitivities of our parameters on the value of *R*_0_ by calculating the PRCC with respect to equation (3.6)—we base our calculation of PRCC on methods used in prior studies [60]. We find that parameters related to an interaction with the environmental reservoir (the population of needles) such as *α* and *β*, the couplings between hosts and needles, are at least as central to HCV dynamics as parameters traditionally associated with an epidemic, such as *π*_*S*_, the birthrate of susceptibles, *μ*, the combined death and cessation rate of PWID, and *τ*, the rate of progressing to treatment (figure 3). This fortifies the notion that W.A.I.T.-specific properties dictate the spread of HCV, providing opportunities to explore more precise targeting by public health interventions.

### 3.5. HCV W.A.I.T. model and simulated interventions: needle exchange programmes

Having demonstrated the relevance of injection drug equipment in terms of how it influences the basic reproductive number, we can consider the utility of the model with respect to other properties, including how it offers insight into potential interventions.

**Figure 4.**
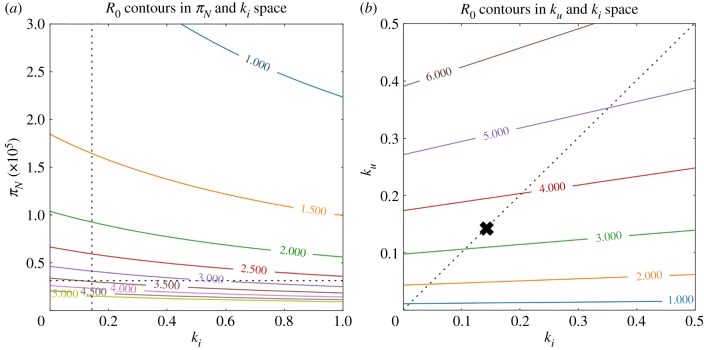
HCV *R*_0_ as a function of various model features. (*a*) The relationship between the rate of acquisition of clean needles *π*_*N*_ and the discard rate of infected needles *k*_*i*_ with respect to various values of *R*_0_. The curves are contours of *R*_0_ and are labelled by the associated *R*_0_ value. The vertical and horizontal dashed lines indicate the chosen values for their respective parameters (we fix *k*_*u*_ to the value specified in table 1). (*b*) The relationship between the infected and uninfected needle discard rate, with respect to *R*_0_. The diagonal line represents where *k*_*u*_ = *k*_*i*_. The ‘*x*’ indicates the value chosen for *k*_*u*_ and *k*_*i*_ in the model (we set *k*_*u*_ = *k*_*i*_ in the model). Notice that moving upwards along this diagonal increases *R*_0_. (Online version in colour.)

In figure 4*b*, we demonstrate how changing *k*_*u*_ and *k*_*i*_ modifies the value of *R*_0_. Notice that *R*_0_ is reduced by increasing *k*_*i*_ across fixed values of *k*_*u*_, and the opposite effect—increasing *R*_0_—is observed when increasing *k*_*u*_ along fixed values of *k*_*i*_. That is, removing infected needles at an increased rate may decrease infection risk in a population of PWID, while removing uninfected needles can increase the risk. One can also see that increasing *k*_*u*_ and *k*_*i*_ simultaneously, along the dashed line—where *k*_*u*_ = *k*_*i*_—will increase *R*_0_. This suggests that if a distinction between infected and uninfected needles cannot be established (as is often the case) then discarding needles indiscriminately can potentially exacerbate the spread of the infection.

This is a result of the fact that when *k* := *k*_*u*_ = *k*_*i*_, the expression for *R*_0_ is proportional to

3.8 $$\sqrt{\frac{k}{\epsilon +k}}\;.$$

We highlight this to show the explicit dependence on *k* in the *R*_0_ expression. Notice that this factor is monotonically increasing in *k*, indicating that no matter the values of other parameters in the model, as long as they are all positive, *R*_0_ will necessarily *increase* with *k*. Notice also that when ϵ = 0, the *k* dependence cancels out entirely. This indicates that when ϵ = 0, meaning that there is no flow of infected needles back to the uninfected compartment, then *R*_0_ is not modified by the discard rate of needles. We point the reader to the electronic supplementary material, appendix, for a more thorough discussion of this point.

**Figure 5.**
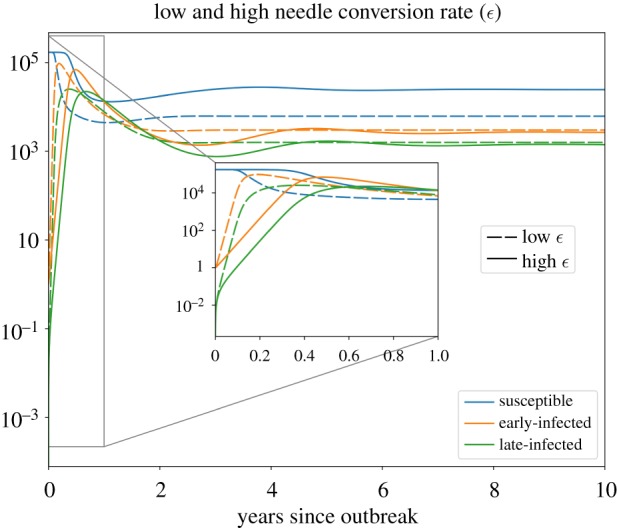
The dynamics of susceptible (blue), early-infected (orange) and late-infected (green) populations in two parameter regimes: high and low ϵ, the conversion rate of needles from infected to uninfected. The solid lines represent the dynamics for $\epsilon =2\;{\mathrm{day}}^{-1}$ (high ϵ), and dashed lines are the dynamics for $\epsilon =0.33\;d^{-1}$ (low ϵ). In the high-ϵ regime, we find that the susceptible population at equilibrium is ≈4 times that of the low-*ε* regime, and the infected populations are each ≈89% of their low-ϵ counterparts at equilibrium (note the log scale on the *y*-axis). (Online version in colour.)

Next, we considered how certain interventions can modify the transfer rate of needles from infected to uninfected states, through modifying the ϵ parameter in our study (figure 5). A high ϵ value would indicate a scenario where needles move quickly from an infected state to an uninfected state. This would apply to settings where viral decay on a needle is high, or when infected needles are directly exchanged for uninfected ones (as in certain needle exchange programmes). The model is run with all uninfected populations initialized at their DFE values (*S* = 170 000 and *N*_*u*_ = 220 000), and we initialize *I*_*E*_ = *N*_*u*_ = 1, and *I*_*L*_ = 0. In the high ϵ scenario, we observe generally slower dynamics and higher overall susceptible population sizes, along with lower infected populations (on long time scales).

## 4. Discussion

While diseases transmitted through injection drug use have been the object of prior modelling efforts, none have specifically investigated how injection equipment plays a role in the dynamics of HCV. Prior models of injection equipment have focused on HIV [24,25], and/or been so complicated that their structure is not easily translated to any other settings [23]. In this study, we model HCV as an indirectly transmitted infection, where the injection equipment is modelled as the environmental reservoir, just as a water source might be modelled in a waterborne infection [8,19]. We label our approach as the W.A.I.T. model, one that incorporates features of other approaches to studying environmentally transmitted pathogens [6,11], but grounding them in a flexible model that can be neatly applied to HCV. Our approach offers several specific insights. For example, we demonstrate that the composite *R*_0_ that defines the entire dynamical system is the geometric mean of *R*_0_ values used to describe each of two sub-components: disease flow through the hosts and flow through the injection equipment (equation (3.7)). This observation offers a practical suggestion for studying diseases like HCV: epidemiologists and modellers must understand, through empirical studies, properties of all major actors in the system (hosts and environmental injection drug equipment in the case of HCV).

The mathematical model of HCV presented in this paper (described as a W.A.I.T. model; see §§2 and 3) also offers nuanced findings about the dynamics of disease. Firstly, our model highlights the differing roles of uninfected and infected injection on disease dynamics. Specifically, the model speaks to the potential utility of harm reduction policies: indiscriminately removing injection equipment from a system—without an overall shift in needle populations from infected to uninfected—might increase the rate of infection. In order to attenuate an epidemic, intervention strategies should focus on steering the population of needles towards being more uninfected. Therefore, ideal intervention efforts should aim to decrease sharing events on an infected needle. This helps to explain why programmes like safe injection might be effective [61]: they do not change the number of infected needles in the system directly, but can alter the sharing rate, and consequently, the probability of sharing an infected needle.

Finally, understanding the dynamical properties of disease transmitted through injection drug use is now especially relevant as a result of the modern opioid epidemic. This epidemic is typified by recreational use of prescription and illicit opioids, with injection drug use being a major route through which drugs are consumed [42]. The relevance of viral diseases among opioid users gained national attention during a 2015 outbreak of HIV in rural Indiana that was driven by an injected opioid called oxymorphone [62,63]. This outbreak raised alarms in the public health community, and officials are increasingly aware of the potential for future outbreaks. However, it was not until relatively recently that the role of the opioid crisis in HCV transmission has been examined [64,65]. We propose, in closing, that modelling approaches (in general, and not relegated to the methods proposed in this study) are crucial for understanding, attenuating or preventing explosive outbreaks of HCV in an age when a new opioid epidemic has emerged.

## Supplementary Material

Supplemental Appendix

## Data accessibility

Code for the mathematical models presented in this paper are available on GitHub (https://github.com/ogplexus/WAIT).
