# Supplementary material for: Hepatitis C virus modelled as an indirectly transmitted infection highlights the centrality of injection drug equipment in disease dynamics
Source: J R Soc Interface. 2019 Sep 4;16(158):20190334. doi: 10.1098/rsif.2019.0334 (PMC6769301; doi:10.1098/rsif.2019.0334)
Supplement: Supplemental Appendix [file rsif20190334supp1.pdf]

Supplemental Appendix

Hepatitis C virus modeled as an indirectly-transmitted infection highlights the centrality of injection drug equipment in disease dynamics

Miles D. Miller-Dickson[1,◐] & Victor A. Meszaros[1,◐],
Salvador Almagro-Moreno[2,3], C. Brandon Ogbunugafor[1,ϟ]

[1] *Department of Ecology and Evolutionary Biology – Brown University, Providence RI, 02906 USA*
[2] *Burnett School of Biomedical Sciences – University of Central Florida, Orlando, FL, 32827 USA*
[3] *National Center for Integrated Coastal Research – University of Central Florida, Orlando, FL, 32816 USA*

◐ These authors contributed equally to this work
ϟ Corresponding Author: brandon_ogbunu@brown.edu

The Hepatitis C Virus WAIT Model

Additional notes on the structure of the model

Here we provide justification for two terms in the model that would benefit from elaboration: (a) the rate of new infection of susceptible individuals, $\beta S \frac{N_i}{N_i+N_u}$, and (b) the rate of new infection of clean needles, $\alpha(I_E + I_L)\frac{N_u}{N_i+N_u}$; other terms in the model are fairly generic.

For (a), let us call the per capita injection rate γ, and the probability that a needle sheds enough of a viral load to render a host infected δ. Then, γS represents the daily rate that susceptible hosts are injecting drugs. In our model, we homogenize the populations of needles and hosts by assuming that the likelihood of injecting with an infected needle is in proportion to the *fraction* of infected needles in circulation $\frac{N_i}{N_i+N_u}$. Thus, the rate at which susceptible hosts inject with infected needles is $\gamma S \frac{N_i}{N_i+N_u}$. Lastly, the fraction of these injections where enough of a viral load renders the host infected is δ, and so the total rate of new host infections due to infected needles is given by $\gamma\delta S \frac{N_i}{N_i+N_u}$. For simplicity, in our model, we set $\gamma\delta = \beta$.

For (b) (the rate of new infection of clean needles via infected hosts) we suppose that the rate at which needles are infected is proportional to the number of infected individuals and is scaled by the daily rate of injections per capita, γ. The product, $\gamma(I_E + I_L)$ represents the average number of injections per day by all infected individuals. We assume that the fraction of these injection events with *uninfected* needles is given (approximately) by the fraction of uninfected needles in circulation, $\frac{N_u}{N_i+N_u}$, so $\gamma(I_E + I_L) \times \frac{N_u}{N_i+N_u}$ represents the rate of injections with the potential to infect a needle. The fraction, call it ζ, of these injection events that shed enough of a viral load to render a needle infected scales this rate, giving $\gamma\zeta(I_E + I_L)\frac{N_u}{N_i+N_u}$. For simplicity, we set $\gamma\zeta = \alpha$.

Note that this approach homogenizes the populations of needles and hosts in the sense that it assumes everyone in the population of people who inject drugs (PWID) exchanges with the *same* environmental reservoirs—the infected and uninfected needle populations. We assume homogeneity, although in a setting with severely skewed distributions of needles among users' collections or in communities with wildly varying sharing practices, this approximation will be a poor one.

Analytic calculation of $\mathrm{R_0}$

Below, we provide the T and Σ matrices used to calculate R_0. In this text, we follow the lines of [1], which establishes an algorithm for calculating R_0 as the maximum eigenvalue—also called the *spectral radius*—of the matrix $-T \cdot \Sigma^{-1}$. This matrix is sometimes cited as the *Next Generation Matrix*, however, as Diekmann et. al. (2009) [1] point out, this matrix may actually be larger than the true next generation matrix. Nevertheless, the maximum eigenvalue of $-T \cdot \Sigma^{-1}$ will be the same as that of the true next generation matrix. The steps to construct T and Σ are outlined in [1]. Essentially $T + \Sigma = J$, where

J is the Jacobian of the infected subsystem—comprised of the infected compartments of the ODE system; I_E, I_L, and N_i, in this case—calculated at the disease free equilibrium (DFE). T refers to terms in the Jacobian related to the transmission of *new* disease in the system, i.e. the conversion of uninfected compartments to infected ones. Σ therefore contains all other terms in the Jacobian. For the HCV system, one finds the following.

$$T = \begin{pmatrix} 0 & 0 & \frac{\beta k_u}{\mu}\frac{\pi_S}{\pi_N} \\ 0 & 0 & 0 \\ \alpha & \alpha & 0 \end{pmatrix}$$

$$\Sigma = \begin{pmatrix} -(\omega+\tau+\mu+\phi) & 0 & 0 \\ \omega & -(\mu+\tau) & 0 \\ 0 & 0 & -(\epsilon+k_i) \end{pmatrix}$$

From this one can construct $-T \cdot \Sigma^{-1}$:

$$-T \cdot \Sigma^{-1} = \begin{pmatrix} 0 & 0 & \frac{\beta k_u \pi_S}{\mu(\epsilon+k_i)\pi_N} \\ 0 & 0 & 0 \\ \frac{\alpha(\mu+\tau+\omega)}{(\mu+\tau+\phi+\omega)(\mu+\tau)} & \frac{\alpha}{\mu+\tau} & 0 \end{pmatrix}$$

From this, one can calculate the maximum eigenvalue of $-T \cdot \Sigma^{-1}$. One finds,

$$R_0 = \sqrt{\frac{\alpha\beta k_u \pi_S(\mu+\tau+\omega)}{\pi_N \mu(\mu+\tau)(\mu+\tau+\phi+\omega)(\epsilon+k_i)}}$$

The reproductive ratio is generally interpreted as the average number of secondary infections per capita caused by infected hosts in the time that these hosts have the infection, when the system is near the *disease-free* equilibrium (DFE). In the context of the WAIT modelling scheme, the interpretation of the reproductive ratio may be modified somewhat as the spread of infection is mediated through interactions between living hosts and an environmental intermediate. In this framework, does the R_0 value represent the number of new infections within the environment or within the population of living hosts? It will turn out that the R_0 value in the WAIT framework represents a kind of average of both of these interpretations, or more precisely, a geometric mean of the two. To put this concretely, we look at the HCV R_0 formula from a perspective that illuminates its nature as a geometric mean.

There are two modes of transmission of new infection in the HCV model. One is the infection of clean needles due to injection by infected hosts, and the other is the infection of susceptible hosts due to injection by infected needles. These two modes of transmission each have a reproductive ratio associated with them: the first would be the number of new infected needles caused by a single infected host in a fully susceptible population of needles, in the average amount of time that a host is infected. The second would be the number of new infections of susceptible hosts caused by a single infected needle in fully susceptible population of hosts, in the time that the needle is infected. The first of these two, which we will denote by X, can be derived by considering the rate of transmission of infection to clean needles, per infected host. In the dynamical equations, the total rate of the infection of needles is given by $\alpha(I_E + I_L)\frac{N_u}{N_i+N_u}$, hence the rate *per infected host* is simply $\alpha\frac{N_u}{N_i+N_u}$. At the disease-free equilibrium, the fraction $\frac{N_u}{N_i+N_u}$ is unity since there are no infected needles in the population. This leaves α as the per capita rate of new infection of needles. There are two infected compartments that can contribute to new infection of needles, I_E and I_L, and a host enters the early-stage compartment first before potentially progressing (with rate ω) to the late-stage. The number of new infections will be therefore be the sum of the infections of needles caused by a host moving through both infected stages, and we will have to weight the number of infections from the I_L compartment by the fraction of the time a host progresses to this stage. The average time that a host spends in the I_E compartment is $1/(\mu+\tau+\phi+\omega)$—i.e. the reciprocal of the exit rate of that compartment. The average amount of

time a host spends in the I_L compartment is $1/(\mu + \tau)$. Thus, since α represents the per capita rate of new infection of needles by infected hosts, then $\alpha \times 1/(\mu+\tau+\phi+\omega)$ is the average number of new infections of needles by hosts in the I_E compartment. Whereas, $\alpha \times 1/(\mu + \tau)$ is the number of new infections of needles caused by hosts in the I_L compartment. The fraction of the time a host progresses from I_E to I_L is given by $\omega/(\mu+\tau+\phi+\omega)$, since this represents the fraction of the rate of exiting the I_E compartment by exactly *one* of the four possible ways; namely, by progressing to I_L, as opposed to death/cessation (μ), treatment (τ), or spontaneous recovery (ϕ). Thus, the average number of needle infections caused by an infected host is given by $\frac{\alpha}{\mu+\tau+\phi+\omega} + \frac{\alpha\omega}{(\mu+\tau)(\mu+\tau+\phi+\omega)}$. The first term represents the rate of new infections of needles caused by hosts in the I_E compartment, multiplied by the time spent in that compartment, and the second is the same but for the I_L compartment, and is scaled by the fraction of time hosts progress to the I_L stage. Simplifying somewhat, X is given by $X = \frac{\alpha(\mu+\tau+\omega)}{(\mu+\tau)(\mu+\tau+\phi+\omega)}$.

Note that we use the reciprocal of the rate terms as the average time spent in a compartment. That is, if the average time an agent spends within a compartment, such as I_E, is 5 days, for example, then one would expect 1/5 of the compartment to leave daily. This is because one expects that on any day, 1/5 of the compartment's population is comprised of agents who entered the set 5 days ago, 1/5 is comprised of those who entered 4 days ago, and so on—here the assumption is that the time spent in a compartment, as well as the entrance rate into the compartment, each exhibit little variance over time. So, the average time within a compartment is the reciprocal of the exit rate—where the exit rate is taken as a *proportion* of the total population, as opposed to an *absolute* rate of change in the population size.

The other reproductive ratio Y can be derived similarly by considering the rate of new host infections due to infected needles: $\beta S \frac{N_i}{N_i+N_u}$. First, in the disease free equilibrium the value for S^* is given by π_S/μ. Second, near the disease-free equilibrium $N_i << N_u$ and hence we can approximate the fraction: $\frac{N_i}{N_i+N_u} = \frac{N_i}{N_u}\frac{1}{\frac{N_i}{N_u}+1} \approx \frac{N_i}{N_u}(1 - \frac{N_i}{N_u}) \approx \frac{N_i}{N_u}$, neglecting the $\left(\frac{N_i}{N_u}\right)^2$ term. The result is that near the DFE, the rate term $\beta S \frac{N_i}{N_i+N_u}$ can be expressed as $\beta\left(\frac{\pi_N}{\mu}\right)\frac{N_i}{N_u}$. The DFE value for N_u—the uninfected needles—is given by π_N/k_u, which is easily verified by inspection of the N_u dynamical equation. Thus, inserting this into the above rate, and considering the rate *per infected needle* N_i, one is left with $\beta\left(\frac{\pi_N}{\mu}\right)\left(\frac{k_u}{\pi_N}\right)$ as the rate of new host infections caused by infected needles per infected needle. As in the prior argument, the average lifetime a needle spends infected is given simply by the reciprocal of the exit rate for agents in the set. In this case, the exit rate of infected needles is given by ϵ—the virus decay rate—and by k_i—the infected needle discard rate. Hence, the average lifetime for an infected needle is given by $1/(\epsilon + k_i)$. Thus, Y is given by $\beta\left(\frac{\pi_N}{\mu}\right)\left(\frac{k_u}{\pi_N}\right) \times 1/(\epsilon + k_i) = \frac{\beta\pi_N k_u}{\mu(\epsilon+k_i)\pi_N}$. One will notice that the equation above for R_0 can be written as

$$R_0 = \sqrt{\frac{\alpha(\mu+\tau+\omega)}{(\mu+\tau)(\mu+\tau+\phi+\omega)}} \times \sqrt{\frac{\beta k_u \pi_S}{\mu(\epsilon+k_i)\pi_N}} = \sqrt{XY}$$

In other words, the R_0 can be viewed as the geometric mean of the two quantities X and Y discussed above.

The dynamics of needles

Our model allows us to distinguish between the discard rates of infected and uninfected needles. However, while it may be that interventions such as needle-exchange programs are capable of increasing the infected needle discard rate above that of the uninfected needles, in many circumstances, the distinction between the two might be more difficult to disentangle. Thus, in many cases there may not be a discernible distinction between k_u and k_i, in which case these rates are effectively equal—we will denote this *universal* discard rate by k $(= k_u = k_i)$.

Our model presents a somewhat counter-intuitive dynamic when $k_u = k_i$: Since the infection rate of needles relies on the proportion of uninfected needles in a population, reducing the proportion of clean needles in the population can actually exacerbate an infection. Namely, increasing the discard rate of all needles will, in particular, reduce the number of uninfected needles in the population and—due to asymmetries in how infected and uninfected needles are treated in the model—can actually lead to an increase in the *proportion* of infected needles in the population, increasing the probability that a user will become infected. Asymmetries between infected and uninfected needles include (1) uninfected needles are fueled by a constant source, π_N, whereas infected needles are fueled only by contact with a varying infected source, and (2) *infected* needles can flow back to the uninfected compartment in proportion to the number of needles infected at that instant, effectively augmenting the discard rate of infected needles, k_i, with the additional term ϵ. In this way, there is a kind of biasing towards the flow of needles into the uninfected compartment. That is, the uninfected compartment receives a constant flux

from the π_N term along with an additional flux from viral decay of infected needles, ϵ. The infected compartment, however, will take losses from the viral decay and can only gain from infected needle use. This is only a heuristic description, but it motivates how changes in the needle discard rate, though equal for both compartments, may have differing impacts on their dynamics, and in particular on their steady-state values. We examine the mathematical details of this behavior below.

A public health interpretation of this would simply be removing all needles from the PWID community (through law enforcement, for example) without a proportional increase in safe, clean needles (as supplied in clean-injection sites or needle-exchange programs), potentially intensifying the local HCV epidemic as a result. Figure S1(C) shows the steady state fraction of infected needles in a population of PWID, as a function of this universal discard rate k. Notice that for the parameters chosen in our model, the proportion of infected needles increases when the discard rate k is increased.

In such circumstances, the disease burden and value of R_0 are both increased. In Figure 4b in the main text, we demonstrate how changing k_u and k_i can modify the value of R_0. We find that increasing k_u while moving along a constant value of k_i has the effect of increasing the R_0 value. Whereas, increasing k_i while moving along a constant value of k_u reduces R_0. That is, removing more uninfected needles while keeping the same infected needle discard rate can harm a population of PWID, and doing the opposite helps the population. One can also see that increasing k_u and k_i simultaneously along the diagonal line—where $k_u = k_i$—will increase R_0. This suggests that if a distinction between infected and uninfected needles cannot be determined (as is often the case), then adding clean needles to a population has a larger impact on disease burden than flatly removing all needles.

Equilibrium values of the infected needles

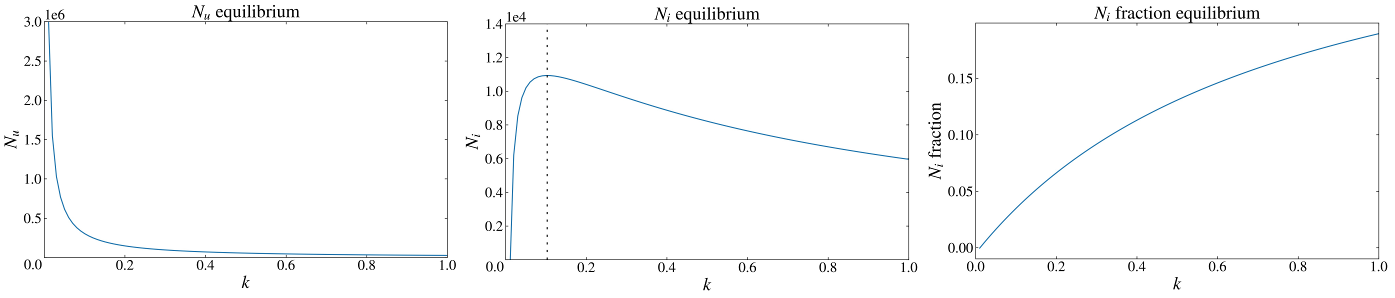

(a) (b) (c)

Fig. S1: **(a)** represents the number of uninfected needles, N_u, as a function of the discard rate, k. **(b)** shows the same but for N_i, the number of infected needles in the population. The vertical dashed line shows the value of k where $dN_i^*/dk = 0$ ($k \approx 0.10$). **(c)** shows the *fraction* of infected needles in the population across the fractional discard rate k, i.e. it shows the value of $N_i/(N_u + N_i)$ at equilibrium as a function of k. Note, that these are endemic equilibria, whereas the disease-free equilibrium will occur when the infection fully clears from the population of PWID, and the population of infected needles goes to zero accordingly.

By setting the model equations to zero, one can determine the equilibrium or steady state values of the agents as a function of the parameters in the model. In doing so, we can demonstrate more quantitatively how the proportion of infected and uninfected needles is affected by modifying the discard rate of needles. In Fig. S1 (a) and (b) we plot the equilibrium values for N_u and N_i as a function of k (where $k = k_u = k_i$).

One will notice that the equilibrium value of N_u is a monotonically decreasing function of k (at least for k between 0 and 1), whereas N_i actually increases with k first before gradually decreasing. That is, whereas the steady state value of uninfected needles always decreases when the universal discard rate k is increased, the steady state value of the infected needles actually has the potential to rise if k is increased from a sufficiently small value. This is consistent with a prior point about the effects of flatly discarding needles, without regard to their infected or uninfected status: it can increase the proportion of infected needles in a population and thus fuel the epidemic. Fig. S1(C) shows the *fraction* of infected needles in the population, and although one finds that the absolute number of infected needles in equilibrium will subside beyond a certain value of k (in our case this is around $k = 0.10$), we find that the *fraction* of infected needles is a monotonically increasing function of k. That is, for the parameters chosen, discarding needles universally will increase the likelihood of encountering an infected needle in the population of PWIDs. We emphasize that this insight was only possible because the needle reservoir (the environment) was modelled separately, a central feature of WAIT.

Below, we provide the results of our analytic calculation of the equilibrium values of the needle populations, N_i^* and N_u^*, as a function of k. We find that both populations can be expressed in a similar form,

$$N_i^*(k) = \frac{x_i + y_i k}{k(A + Bk)}, \qquad N_u^*(k) = \frac{x_u + y_u k}{k(A + Bk)}$$

where the parameters x_i, y_i, x_u, y_u, A, B (given explicitly below) are independent of k and which are functions of the other parameters in the model.

$$\begin{aligned}
x_i &= -\epsilon\mu\pi_N^2(\mu+\tau)(\mu+\tau+\phi+\omega) \\
y_i &= \alpha\beta\pi_S\pi_N(\mu+\tau+\omega) - \mu\pi_N^2(\mu+\tau)(\mu+\tau+\phi+\omega) \\
x_u &= \epsilon\pi_N^2(\mu+\tau)(\beta(\mu+\tau+\omega)+\mu(\mu+\tau+\phi+\omega)) \\
y_u &= \pi_N^2(\mu+\tau)(\beta(\mu+\tau+\omega)+\mu(\mu+\tau+\phi+\omega)) \\
A &= \beta\epsilon\pi_N(\mu+\tau)(\mu+\tau+\omega) \\
B &= \beta(\mu+\tau+\omega)(\alpha\pi_S+(\mu+\tau)\pi_N)
\end{aligned}$$

Given the similar form that the infected and uninfected needle populations have at equilibrium, we can calculate their derivatives with respect to k simultaneously. Using N, x, and y to refer generically to one of the needle populations at equilibrium and its corresponding coefficients in the equilibrium formula, one finds that.

$$\frac{dN}{dk} = \frac{d}{dk}\left(\frac{x + yk}{k(A + Bk)}\right) = -\frac{(A + 2Bk)x}{k^2(A + Bk)^2} - \frac{By}{(A + Bk)^2}$$

from this it follows that $dN/dk = 0$ when $-(A + 2Bk)x = Byk^2$ or that,

$$k_\pm = \frac{-Bx \pm \sqrt{(Bx)^2 - ABxy}}{By}$$

For the parameters chosen in the model, we find that both k_+ and k_- are negative when $(x, y) = (x_u, y_u)$ and when $(x, y) = (x_i, y_i)$ only k_+ gives a positive value, namely $k_+ \approx 0.1032$. This result can be seen in figure S1, where one will notice that $dN_u/dk < 0$ for all values of k, whereas $dN_i/dk = 0$ at $k \approx 0.1032$. k_+ represents a threshold value for the discard rate of needles. Above this value, the total number of infected needles at equilibrium can be decreased if k were to be increased, and below this value, raising k will only increase the total number of infected needles at equilibrium. This result is counter-intuitive but it can be understood by considering the fact that increasing k means that more needles *of all types* are removed from the system in a given amount of time. In particular, if this leads to a sufficient reduction of the uninfected needles in circulation then, since infection rates depend on the *fraction* of both types of needles in the model, it is possible to reduce the fraction of uninfected needles in the population, thereby increasing the fraction of infected needles and increasing the infection rates among hosts. This would in turn lead to a higher total number of infected needles in equilibrium. We also find that for the parameters chosen, the *fraction* of infected needles at equilibrium is monotonically increasing with k, at least for the range of k considered in this paper (Figure S1 (c)).

If we examine the infected needle fraction more closely, we can quantify the extent to which increasing k will *increase* the fraction of infected needles at equilibrium. We will use n_i^* to refer to the infected needle fraction at equilibrium, viz. $n_i^*(k) = N_i^*/(N_i^* + N_u^*)$.

$$n_i^*(k) = \frac{\frac{x_i + y_i k}{k(A+Bk)}}{\frac{x_i + y_i k}{k(A+Bk)} + \frac{x_u + y_u k}{k(A+Bk)}} = \frac{x_i + y_i k}{(x_i + x_u) + (y_i + y_u)k}$$

assuming that $k(A+Bk) \neq 0$. For simplicity, we will set $x = x_i + x_u$ and $y = y_i + y_u$. In order to determine how increasing the discard rate k can increase the infected needle fraction, we calculate dn_i^*/dk.

$$\frac{dn_i^*}{dk} = \frac{y_i x - y x_i}{(x+yk)^2}$$

Suppose $dn_i^*/dk > 0$, then this is equivalent to the following constraint,

$$y_i x - y x_i > 0.$$

Using the definitions given above for $x_i, y_i, x,$ and y, one can identify a simple expression for $y_i x - y x_i$:

$$\alpha\beta\epsilon\pi_N^3\pi_S(\mu+\tau)(\mu+\tau+\omega)(\beta(\mu+\tau+\omega)+\mu(\mu+\tau+\phi+\omega))$$

One notices that the above expression trivially satisfies the inequality since all parameters in the model are positive. This indicates that our model predicts that the *fraction* of infected needles will increase with k regardless of the other parameters in the model, so long as they remain positive.

$\mathbf{R_0}$ *Sensitivity*

We employ a sensitivity analysis for two purposes: (i) to establish that model dynamics do not rely on the particular values of any one parameter and (ii) that the sensitivity of the dynamics is *shared* fairly evenly among the parameters. This is an indication that when the phenomena of interest are modelled in this manner, one can expect that the dynamics of the infection are also shared fairly evenly among the parameters.

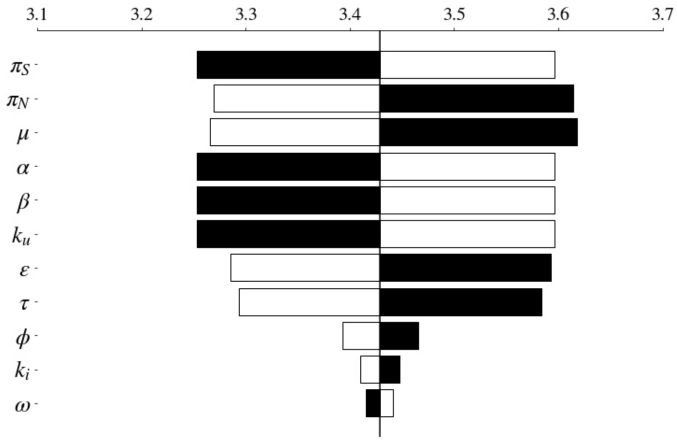

Fig. S2: **$\mathbf{R_0}$ Tornado**: A tornado diagram for parameters in the R_0 formula. Black bars indicate the value of R_0 when the associated parameter is *decreased* by 10% from the value chosen in the model. White bars indicate the value of R_0 when the associated parameter is *increased* by 10%.

Fig. S2 demonstrates how the value of R_0 *shares* its dependence evenly among most of the parameters. We calculated the *Partial Rank Correlation Coefficient* (PRCC) with respect to the value of R_0, for each of the parameters in the R_0 formula (Figure 6 in the main text). The calculation followed the lines given in Blower and Dowlatabadi (1994) [2], and we will briefly recapitulate the calculation here.

There are 11 parameters in the R_0 formula (equation (3.6) in the main text). Using Latin Hypercube Sampling (LHS), 100 samples of the 11 parameters were taken. Each sample of 11 parameters was used to calculate a value of R_0. Then, the resulting R_0 values and parameters were ranked according to their value among the 100 samples. That is, the R_0 value and the 11 parameters in each sample were assigned numbers 1–100 depending on how they ranked compared to other samples. This results in 11 vectors, and one additional vector for R_0, of length 100, whose entries are just some ordering of the whole numbers between 1 and 100—we will call them *rank* vectors. Then, between any two of the 11 rank vectors and one additional rank vector for R_0 we can calculate the generic correlation coefficient for the 100 samples. If we arrange the parameters, indexing them 1 through 11, and the R_0 value, giving it the index 12, into a list of *variables*, then we can construct the correlation coefficients C_{ij}, between the ith and jth variable, into a symmetric matrix C.

$$C_{ij} = \frac{\sum_{k=1}^{100}(r_{ik} - \mu)(r_{jk} - \mu)}{\sqrt{\sum_{k=1}^{100}(r_{ik} - \mu)^2 \sum_{k=1}^{100}(r_{jk} - \mu)^2}}$$

r_{ik} in the equation above is the rank of the ith variable (note that R_0 is included in the variables) in the kth sample, and $\mu = (1 + 100)/2 = 50.5$ is the average rank. Thus, for i and j between 1 and 11, C_{ij} is the correlation coefficient between the ith and jth parameter, and for i between 1 and 11, $C_{i,12}$ $(= C_{12,i})$ are the rank-correlations between the ith parameter and the value of R_0. Note, that the diagonal values of C are all one. Next, we construct the matrix B, which is simply the matrix inverse of C, i.e. $B = C^{-1}$. Lastly, the PRCC value for the ith parameter is constructed from the matrix elements of B according to the following formula,

$$PRCC_i = \frac{-B_{i,n+1}}{\sqrt{B_{ii}B_{n+1,n+1}}}$$

where $n = 11$—the number of parameters in the R_0 formula. This entire calculation was repeated 50 times, with 50 sets of 100 LHS samples. The values shown in the PRCC calculation (Main text, Figure 3) show the average over these 50 iterations, with the standard deviations as error bars.

REFERENCES

[1] O. Diekmann, J. A. P. Heesterbeek, and M. G. Roberts. The construction of next-generation matrices for compartmental epidemic models. *Journal of the Royal Society Interface*, 7:873–885, 2010. doi:10.1098/rsif.2009.0386.

[2] S. M. Blower and H. Dowlatabadi. Sensitivity and uncertainty analysis of complex models of disease transmission: An model, as an example. *International Statistical Review*, 62(2):229–243, August, 1994.
